# Training dual-task walking in community-dwelling adults within 1 year of stroke: a protocol for a single-blind randomized controlled trial

**DOI:** 10.1186/1471-2377-12-129

**Published:** 2012-10-31

**Authors:** Prudence Plummer-D’Amato, Anastasia Kyvelidou, Dagmar Sternad, Bijan Najafi, Raymond M Villalobos, David Zurakowski

**Affiliations:** 1 Department of Physical Therapy, Northeastern University, 360 Huntington Ave, 6 Robinson Hall, Boston, MA, USA; 2 Department of Biology, Northeastern University, Boston, MA, 02115, USA; 3 Departments of Biology, Electrical and Computer Engineering, and Physics, Northeastern University, Boston, MA, 02115, USA; 4 Department of Surgery, University of Arizona, Tucson, AZ, 85724, USA; 5 Arizona Center on Aging, University of Arizona, Tucson, AZ, 85724, USA; 6 New England Rehabilitation Hospital, Woburn, MA, 01801, USA; 7 Departments of Anesthesia and Surgery, Children’s Hospital, Boston, MA, 02115, USA; 8 Departments of Anesthesia and Biostatistics, Harvard Medical School, Boston, MA, 02115, USA

**Keywords:** Stroke, Gait, Dual-task, Attention, Cognition, Rehabilitation, Obstacle avoidance, Participation, Physical therapy

## Abstract

**Background:**

Community ambulation is a highly complex skill requiring the ability to adapt to increased environmental complexity and perform multiple tasks simultaneously. After stroke, individuals demonstrate a diminished ability to perform dual-tasks. Current evidence suggests that conventional rehabilitation does not adequately address gait-related dual-task impairments after stroke, which may be contributing to low levels of participation and physical inactivity in community-dwelling stroke survivors. The objective of this study is to investigate the efficacy of dual-task gait training in community-dwelling adults within 1 year of stroke. Specifically, we will compare the effects of dual-task gait training and single-task gait training on cognitive-motor interference during walking at preferred speed and at fastest comfortable speed (Aim 1), locomotor control during obstacle negotiation (Aim 2), and spontaneous physical activity (Aim 3).

**Methods/Design:**

This single-blind randomized controlled trial will involve 44 individuals within 12 months of stroke. Following baseline evaluation, participants will be randomly allocated to single- or dual-task gait training. Both groups will receive 12, 30-minute sessions provided one-on-one over 4–6 weeks in an outpatient therapy setting. Single-task gait training involves practice of gait activities incorporating motor relearning principles. Dual-task gait training involves an identical gait training protocol; the critical difference being that the dual-task gait training group will practice the gait activities while simultaneously performing a cognitive task for 75% of the repetitions. Blinded assessors will measure outcomes at baseline, post-intervention, and 6 months after completion of the intervention. The primary outcome measure will be dual-task effects on gait speed and cognition during unobstructed walking. Secondary outcomes include spatiotemporal and kinetic gait parameters during unobstructed single- and dual-task walking at preferred and fastest comfortable walking speeds, gait parameters during high and low obstacle crossing, spontaneous physical activity, executive function, lower extremity motor function, Timed Up and Go, balance self-efficacy, number of falls, and stroke-related disability. Hypotheses for each aim will be tested using an intention-to-treat analysis with repeated measures ANOVA design.

**Discussion:**

This trial will provide evidence to help clinicians make decisions about the types of activities to include in rehabilitation to improve dual-task walking after stroke.

**Trial registration:**

ClinicalTrials.gov NCT01568957

## Background

The achievement of community ambulation after stroke is critical for active participation in everyday activities, preventing social isolation and depression, and enhancing quality of life [1,2]. In the period following discharge, many stroke survivors report low levels of participation in activities outside the home, with walking ability being a major factor influencing community reintegration [3]. Functional community ambulation requires an ability to perform cognitive tasks while walking, and an ability to adapt to extrinsic environmental factors that increase the complexity of mobility, such as obstacle avoidance (e.g., curbs) and time-critical tasks (e.g., crossing the street within the time constraints imposed by traffic signals) [4]. A reduced capacity for dual-task walking and/or limited ability to adapt to changes in environmental context may substantially restrict the degree to which a person is able to participate in his/her life roles.

After stroke, performance of a cognitive task concurrently with walking results in a profound reduction in gait speed [5-7], with corresponding effects on stride duration [5,8,9], stride length [5,6], double limb support time [7,10], and cadence [5,11] (referred to as *dual-task interference,* or *cognitive-motor interference*). Diminished capacity to walk with adequate speed while performing a cognitive task may increase disability in the community or lead to curtailing of participation in everyday activities. Although dual-task performance can also affect the cognitive task [6,11], most research indicates that patients with stroke prioritize the cognitive task, sacrificing gait performance [5,6,8,9].

Current evidence suggests that conventional rehabilitation does not adequately address gait-related dual-task impairments after stroke. Although Cockburn and colleagues [9] found that 7 out of 10 patients showed a reduction in dual-task cost on stride duration after customary rehabilitation, most patients continued to exhibit considerable dual-task interference during walking at discharge, as well as simultaneous interference in the cognitive task. Specific training in dual-tasking may be necessary to improve dual-task walking in people with stroke. A reduction in dual-task interference may be achieved via at least two possible mechanisms: (1) improving automatization of walking by repetitive practice; gait automaticity reduces the attentional requirements of gait, thereby increasing the capacity to perform simultaneous cognitive tasks; and (2) improving dual-task coordination by task-specific training; targeted practice of gait-related dual-tasks may improve performance in these activities. Theoretically, DTGT should result in greater improvement in dual-task performance (reduced dual-task cost) than STGT; however, both may act to increase gait automatization [12,13], since both approaches involve repetitive practice of walking. There is mounting evidence of the value of task-specific training in neurological rehabilitation [14,15]. This evidence indicates that practice should be task-specific and relevant to the patient and context. Based on the principles of task-specific training, practice of dual-task activities during locomotion in a variety of environmental contexts is needed to improve dual-task performance.

Although dual-task training in older adults has been shown to have promise [13,16], there has been little research on dual-task training in stroke. In a randomized controlled trial, Yang et al. [17] compared a motor-motor dual-task intervention (walking while manipulating either one or two balls of various size) to a no-intervention control. Compared to 12 patients who did not receive any intervention, the 13 patients who received dual-task training significantly improved their gait speed during both single-task and dual-task (tray carrying) walking. Our pilot data [18] provide preliminary evidence that a cognitive-motor dual-task intervention can reduce dual-task interference in gait and increase community participation in community-dwelling stroke survivors. However, due to absence of a control intervention in either study, it is not possible to know whether improvements were due to repetitive walking practice (i.e., gait automatization) or the inclusion of dual-task activities (i.e., task-specific training in dual-task coordination). Research is needed to directly compare dual-task gait training to more traditional, single-task gait training approaches in order to identify the optimal parameters of gait rehabilitation that can improve walking and reduce dual-task interference after stroke.

The primary aim of this study is to investigate the effects of dual-task gait training (DTGT) compared to single-task gait training (STGT) on dual-task interference in community-dwelling adults within one year of stroke. Our hypothesis is that DTGT mediates the coordination of dual-tasks and will reduce cognitive-motor interference more than STGT. By measuring dual-task effects in both gait and cognitive tasks, we will be able to examine whether the intervention affects the voluntary allocation of attentional resources during dual-task walking. The second aim is to investigate the effect of DTGT on a more ecologically valid attention-demanding task: obstacle avoidance. Although obstacle crossing has been studied in people with stroke [19-23], there have not been any studies investigating whether training in attentionally-demanding tasks can improve locomotor control during obstacle avoidance. The third aim is to examine the effect of the intervention on participation by quantitatively measuring ambulation and physical activity in the participants’ natural environment using innovative, wireless sensor technology. Despite the considerable threat to participation in everyday life posed by diminished dual-task capacity, research has not previously investigated the relationship between dual-task interference and community participation. This study addresses this important question, and will determine whether DTGT increases community participation more than STGT. Knowing whether an intervention has resulted in meaningful change in a person’s ability to participate in the real world is the ultimate indicator of therapeutic effectiveness.

## Methods

### Design

**Figure 1:**
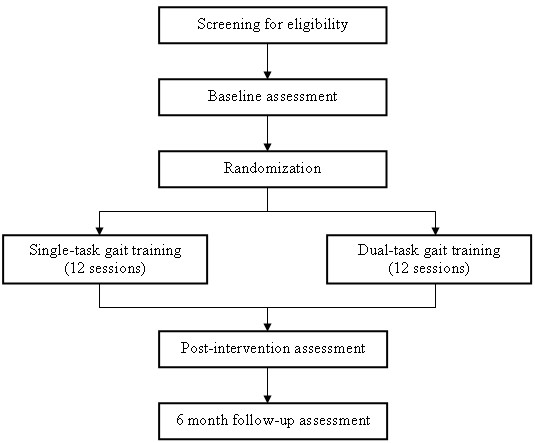
Flow of trial.

This study is a single-blind randomized controlled trial of DTGT versus STGT in an outpatient therapy setting. Following baseline assessment, participants will be randomly assigned to one of the two intervention groups. The primary outcome is dual-task effect on gait speed and cognition (reaction time and accuracy) during unobstructed walking at preferred and fastest comfortable walking speeds. We include the fastest comfortable speed condition to investigate the effects on the intervention on a more attention-demanding gait task. The flow of the trial is illustrated in Figure 1.

### Participants

The participants will be 44 community-dwelling adults within 12 months of stroke. To be included in the study participants must satisfy the following criteria: (a) ≥ 18 years, (b) a diagnosis of stroke consistent with the World Health Organization definition [24] and, where possible, confirmed by CT or MRI, (c) be within 12 months of stroke onset, (d) living in the community, defined as living in one’s home or the home of a friend, relative, or caregiver, (e) able to walk without assistance of another person, (f) self-selected gait speed 0.6 -1.1m/s determined during a 10m walk test, (g) score > 23 on the Montreal Cognitive Assessment (MoCA) [25], which is the cut off at which the sensitivity and specificity of the MoCA are optimized [26]. All participants must be medically stable and approved for participation by the study physician. Additionally, patients must have been discharged from conventional outpatient physical therapy before commencing study interventions. Thus, the patients will be receiving only the study-related physical therapy. Exclusion criteria for the study include: (a) neurological conditions other than stroke, (b) any orthopedic or cardiopulmonary problems limiting gait or physical activity, (c) previous stroke with residual motor deficit, (d) inability to follow a 3-step command, (e) uncontrolled hearing impairment, (f) severe uncontrolled visual impairment, (g) speech-language impairment affecting ability to respond verbally to auditory stimuli, determined by speech-language pathologist, (h) Timed Up and Go test > 15 seconds, (i) lower extremity amputation, (j) not living in the community prior to stroke, and (k) concurrent participation in another clinical trial.

### Randomization and blinding

Following baseline evaluation, the participants will be randomized to either DTGT or STGT using a 1:1 randomization sequence prepared by the study biostatistician. Group assignment will be concealed using opaque envelopes, which will be opened after the completion of the baseline assessment. Research personnel conducting the baseline, post-intervention, and follow-up assessments will be blind to group assignment. The investigator processing the data extracted from activity monitoring will also be blind to group assignment. Participants will be aware of their group allocation, but will be naïve to the study hypotheses. Therapists providing the study interventions cannot be blinded in this study design.

### Interventions

The interventions will be implemented in the outpatient physical therapy clinic at New England Rehabilitation Hospital (Woburn, MA). Physical therapists trained in the study protocols will provide the intervention one-on-one. The two groups (DTGT, STGT) will each participate in a 30-minute training session, 3 times a week for 4 weeks (total of 12 sessions). A maximum of 6 weeks will be allowed to complete the 12 sessions. For participants in both groups, each session will begin with a 5–10 minute warm-up comprising lower extremity stretching, three 10-m walks at self-selected speed and three 10-m walks at fastest speed. The warm-up walks allow the therapist to observe single-task gait performance, (a) as a basis for focusing the goals of the session (e.g. increasing stride length), and (b) to establish a reference of single-task performance for the DTGT group.

#### Single-task gait training

The theoretical framework for the gait training intervention incorporates principles from Gentile’s taxonomy of tasks [27] and the Motor Relearning Program for Stroke [28]. Gentile’s taxonomy of tasks is a model for progressing patients from closed environments (stationary, predictable) to open environments (moving, unpredictable). The Motor Relearning Program for Stroke, from which the task-oriented training approach was derived, advocates 4 steps: (1) task analysis, (2) part-task practice, (3) whole-task practice, and (4) transfer of training to daily life. Using these frameworks, each session will comprise three specific components: (i) part-task practice, (ii) whole-task practice, and (iii) contextual practice to facilitate transfer to real-world environments.

Gait training activities will be performed initially in closed environments (quiet area with few distractions) and progressed as appropriate to an open environment (busy area with unpredictable and moving distractors). Part-task practice includes activities such as dynamic postural control and single-step training; whole-task practice includes activities that involve continuous walking; contextual transfer practice includes obstacle negotiation and outdoor walking, and is an extension of whole-task practice.

In each session, participants will complete 12 repetitions of two part-task and two whole-task practice activities, and 12 repetitions of one contextual transfer activity. To ensure the sessions are the same duration for all participants (30 minutes), after the warm-up, each component will be limited to 10 minutes. For participants with more severe impairments who are not able to complete 12 repetitions of two activities in 10 minutes, therapists will advance to the next component after 10 minutes to ensure that all components of the training program are practiced in each session. Conversely, participants who complete the activities in less than 30 minutes will perform a second set of repetitions of the contextual transfer task. Thus, the total number of repetitions may vary slightly based on individual ability levels, but the exposure time will be identical for all participants.

The gait training activities for each component (part-task, whole-task, contextual transfer) are standardized. Five levels of difficulty are defined for each standardized gait training activity. Participants will progress through the difficulty levels according to individual abilities and the progression guidelines detailed in the Intervention Manual of Procedures.

#### Dual-task gait training

Participants in the DTGT group will practice identical gait activities as the STGT group. The critical difference is that the DTGT group will practice the gait activities while simultaneously performing a cognitive task for 75% of the repetitions. Specifically, in DTGT, the first 3 repetitions will be performed without any added cognitive task, and then 9 repetitions will be practiced under dual-task conditions. Participants in STGT perform all repetitions without any cognitive tasks. A variable-priority training strategy for the dual-task intervention will be used, such that participants will alternate their attentional focus between gait and the cognitive task within each session. Silsupadol et al. have reported the variable-priority approach to be more effective than fixed-priority practice (equal attention to both tasks at all times) and single-task training for improving dual-task gait speed [16], balance (body sway) [13] and cognitive performance (reaction time) [13] in older adults. Thus, we considered variable-priority to be the best available approach for dual-task training at this time.

Ten different cognitive tasks involving a range of cognitive functions (working memory, executive function, visuospatial processing, and language) will be used during DTGT. The sequence of cognitive tasks through the program is standardized to ensure that participants practice an array of cognitive tasks in varying combinations with the gait activities. Two different cognitive tasks will be used in each session. Progressions in difficulty within each cognitive task will be based on individual abilities and will be directed by progression guidelines detailed in the Intervention Manual of Procedures. Participants are not expected to reach the highest level of difficulty for each cognitive task; rather, the task progressions are provided for variation as the participants advance through the program.

### Outcome measures

Primary and secondary outcome measures will be assessed at baseline, after 12 sessions of the intervention, and 6 months following completion of the intervention.

#### Primary outcome measures

The primary outcome measure will be the dual-task effect (DTE) on gait speed and cognition (reaction time and accuracy) during unobstructed walking at preferred walking speed and fastest comfortable walking speed. Dual-task effects will be calculated by dividing the difference between single and dual-task performance by single-task performance, expressed as a percentage [29]. Specifically, for variables in which a higher value indicates better performance (e.g., gait speed, cognitive task accuracy), DTE will be calculated as:

(1) $$\text{DTE}=\frac{\left(\text{dual task}-\text{single task}\right)}{\text{single task}}\times \;100\%$$

Conversely, for variables in which lower values indicate better performance (e.g., reaction time), DTE will be calculated as:

(2) $$\text{DTE}=\frac{-\left(\text{dual task}-\text{single task}\right)}{\text{single task}}\times \;\text{100\%}$$

Thus, for all variables, positive DTE values indicate an improvement in performance in the dual-task condition relative to single-task (i.e., dual-task benefit) and negative DTE values indicate a decrement in performance in the dual-task condition (i.e., dual-task cost).

We will assess dual-task performance in two different dual-task combinations. The two cognitive distractor tasks that will be used in the dual-task conditions will be the auditory Stroop task [30], and the clock task [5]. The Stroop task involves executive function, which is critical in dual-task coordination. Participants will hear the words “high” and “low” spoken in either a high pitch (360 Hz) or a low pitch (180 Hz) and must indicate the pitch of the word they hear (ignoring the actual word presented) as accurately and as quickly as possible. The clock task is a visuospatial reaction time task. Participants will hear a time (e.g., “two-twenty-five”) and respond verbally (yes/no) based on whether the hands of clock for the given time are in the same half or not: “yes” if both hands are in the same half (left/right) of the clock, and “no” if they are not. Visuospatial abilities are highly relevant for navigating the environment. The participants will first practice the Stroop and clocks while sitting. Single-task performance of the cognitive task will then be recorded (sitting), followed by the dual-task conditions (walking at preferred and fastest comfortable speed). No specific instruction regarding task prioritization will be provided for the dual-task trials.

The gait tasks will involve continuous walking for one minute in the laboratory at Northeastern University. Single-task trials at self-selected and fastest comfortable speed will be performed before dual-task trials. Gait speed data will be acquired using a 12 camera Qualisys Motion Capture System. Thirty lightweight reflective markers (12mm) will be placed to anatomical positions of the subject’s lower extremities and pelvis in order to define the respective segments [31,32]. Gait speed will be calculated from the Visual 3D software, and is computed using the actual stride length over the actual stride time.

#### Secondary outcome measures

In addition to gait speed, we will also measure other spatiotemporal parameters of gait (e.g., stride length, double limb support duration) in the single and dual-task conditions described above. Other secondary outcome measures include: spatiotemporal and kinetic gait parameters during high and low obstacle crossing, spontaneous physical activity, executive function, lower extremity motor function, functional gait performance, balance self-efficacy, number of falls, and stroke-related disability.

Spatiotemporal data during obstacle crossing will be acquired as above for unobstructed gait. Additionally, kinetic data will be collected by two AMTI force platforms embedded in the floor of the gait laboratory. Signals from the markers will be digitized at a sampling rate of 100Hz, while raw analog data from the force platforms will be collected at a sampling frequency of 1000Hz. Obstacle crossing will be performed with obstacles at 5% and 15% of leg length. The obstacle will be placed between two force plates along the walkway, enabling force plate data to be acquired before and after the obstacle. We will collect data on step kinematics during obstacle approach to quantify hesitations incurred by the necessary modifications of strides towards the obstacle. In addition, we will measure vertical foot clearance over the obstacle, pre- and post-obstacle clearance distance, ground reaction forces and their variability measured by the force plates, as an indicator of the degree of control of stepping over the obstacle. Using motion capture, we will determine the time profile of the center of mass to examine smoothness of motion progression during obstacle approach and crossing. Since the pre-crossing phase of obstacle negotiation is particularly attention-demanding due to the planning required for step adaptation, this condition allows us to determine the effects of DTGT on a real-world dual-task that has the cognitive load inherently embedded. Participants will complete 4–5 trials in each obstacle condition. Since the obstacle negotiation task itself requires cognitive resources, we will not be assessing obstacle crossing during the Stroop or clock tasks. Rather, the obstacle condition enables direct investigation of a “natural” dual-task.

To measure spontaneous physical activity, participants will wear a physical activity monitor (PAMSys™, Biosensics LLC, MA, USA) embedded in a lightweight breathable T-shirt worn under clothing for two consecutive days after each evaluation. This device uses a combination of miniaturized kinematic sensors housed in a single portable sensor attached to the chest. It can detect body posture (e.g., sitting, lying) as well as provide an accurate assessment of periods of locomotion (e.g., walking, turning), including gait inter-cycle variability during activities of daily living. The validity of this approach has been established in three separate pilot studies and by benchmarking the results with independent analysis by an optical motion system [33-35].

Executive function will be assessed using a computerized Stroop test [36]. DirectRT (Empirisoft, New York, NY) software will record the participants’ reaction times and responses. We will use color-word interference accuracy scores and reaction times to assess executive function at each assessment. Lower extremity motor function will be assessed using the Fugl-Meyer Motor Assessment scale [37]. Functional gait performance will be evaluated using the Timed Up and Go test [38], and the Activities-specific Balance Confidence Scale [39] will measure balance self-efficacy. The Stroke Impact Scale [40] will be used to assess stroke-specific disability. Participants will keep a falls diary between the post-intervention assessment and the 6-month follow-up assessment. Any participant reporting a fall will receive a phone call from the research team to obtain information about the fall and any related injuries.

Additional measures conducted at baseline to further characterize the study sample include: Digit Symbol Modalities Test (speed of processing) [41], Comprehensive Trail Making Test (inhibition of distraction) [42], and the Star Cancellation Test (unilateral spatial neglect) [43], National Institutes of Health Stroke Scale (stroke severity) [44], 6-minute walk test (walking endurance) [45], Melbourne Edge Test (contrast sensitivity), and lower extremity sensation and proprioception. Demographic data including age, time since stroke, medical history, employment status and living situation will also be collected at baseline.

The protocol for this study has been approved by the Northeastern University Institutional Review Board (IRB; #11-06-17) with IRB authorization agreement from New England Rehabilitation Hospital (Woburn, MA). Individuals who wish to participate in this study will provide written informed consent.

### Data analysis

#### Sample size calculation

The study has been powered to detect a minimum difference of 10% in dual-task cost on gait speed between the DTGT and STGT groups. The difference of 10% is based on the established minimal clinically important difference for gait speed decline in older adults (including people with stroke). Perera et al. [46] determined that a decline in gait speed of 0.1 m/s was associated with substantial meaningful change in physical function. Based on the expected mean gait speeds throughout the phases of this study, a dual-task decline of 0.1 m/s corresponds to a 10-12% dual-task cost on gait speed. Assuming within-group standard deviation of 10.15, with 80% power at the 0.05 significance level, we need 18 subjects in each group. Allowing for 20% attrition at the 6-month follow up, we plan to randomize 22 subjects to each group (total 44 subjects).

#### Data analysis plan

DTGT and STGT groups will be compared on baseline characteristics using Student *t*-tests for continuous data and Fisher’s exact test for proportions. The hypotheses will be tested using an intention-to-treat analysis, applying a repeated measures design whereby the two groups will be compared on primary outcomes at: (i) baseline, (ii) after the completion of the intervention, and (iii) 6 months following completion of the intervention. For the primary outcomes of dual-task effects on gait and cognition, a 2 x 3 mixed model repeated measures ANOVA with group (DTGT, STGT) as between-subjects factor and time (pre-training, post-training, 6-month follow-up) as within-subjects factor will be applied to DTE for each gait task separately (preferred walking, fast walking) and for each cognitive task separately (Stroop, clock). A similar analysis will be performed for all secondary outcome measures. Tukey’s honestly significant difference (HSD) post-hoc tests will be applied to examine the significant differences for all ANOVAs. If the normality assumptions for ANOVA are not supported, nonparametric equivalent statistical methods including Mann–Whitney *U*-tests and Wilcoxon signed-ranks tests will be used.

## Discussion

This single-blind randomized controlled trial will determine whether task-specific training in dual-task walking can reduce dual-task interference during gait, compared to single-task gait training. Preliminary studies provide promising evidence that dual-task gait training can improve dual-task gait performance after stroke [17,18]; however, these small studies are limited by the lack of a control group and long-term follow-up. The results of this randomized controlled trial will provide insight into whether reductions in dual-task interference arise from improved automatization of gait, or improved ability to coordinate cognitive and motor tasks performed simultaneously. Therefore, the findings of this study will help clinicians make decisions about the types of activities to include in rehabilitation to improve dual-task walking after stroke.

We will measure the effects of the intervention not only on dual-task gait performance, but also on dual-task cognitive performance, which will reveal information about whether voluntary attention allocation changes over time: for example, whether improvements in dual-task gait performance occur at a cost to dual-task cognitive performance, or whether overall dual-task capacity appears to improve. Secondary outcome measures will provide important information about how DTGT and STGT impact locomotor control during obstacle avoidance, spontaneous physical activity in the community, and other measures of cognitive and motor impairment. This study will make an important and unique contribution to the evidence base for physical therapy rehabilitation post-stroke, because it explores the components of rehabilitation that can potentially minimize disabling dual-task interference in stroke survivors.

## Abbreviations

ANOVA: Analysis of variance; DTE: Dual-task effect; DTGT: Dual-task gait training; IRB: Institutional Review Board; MoCA: Montreal Cognitive Assessment; STGT: Single-task gait training.

## Competing interests

The authors declare that they have no competing interests.

## Authors’ contributions

PP conceived the idea for this study. PP, DS, and BN contributed to the design of the study and obtained the study funding. AK led the development of gait analysis and obstacle negotiation protocols and contributed to writing of this manuscript. RMV contributed to selection of outcome measures and provides patient recruitment and medical oversight. DZ performed sample size calculation and led the development of the statistical analysis plan. PP was primarily responsible for writing this manuscript. All authors assisted in editing. All authors reviewed and approved the final version of this manuscript.

## Pre-publication history

The pre-publication history for this paper can be accessed here:

http://www.biomedcentral.com/1471-2377/12/129/prepub
